# Tumor Necrosis Factor Superfamily 14 Regulates the Inflammatory Response of Human Dental Pulp Stem Cells

**DOI:** 10.3390/cimb46120836

**Published:** 2024-12-11

**Authors:** Abdulelah Alrshedan, Mona Elsafadi, Manikandan Muthurangan, Solaiman Al-Hadlaq

**Affiliations:** 1 Department of Restorative Dental Sciences, College of Dentistry, King Saud University, Riyadh 11545, Saudi Arabia; dr.abdulelah.r@gmail.com; 2 Stem Cell Unit, Department of Anatomy, College of Medicine, King Saud University, Riyadh 11461, Saudi Arabia; melsafadi@ksu.edu.sa (M.E.); mrangan@ksu.edu.sa (M.M.)

**Keywords:** human dental pulp stem cells, TNFSF14, LIGHT, lipoteichoic acid, inflammation, IL 6, IL 8, IL 10, TNF α

## Abstract

Dental caries is a highly prevalent chronic disease that leads to dental pulp inflammation. It is treated by removing the damaged tooth structure and applying a material that promotes resolution of pulpal inflammation. Tumor necrosis factor superfamily 14 (TNFSF14) is an immunomodulatory cytokine and a member of the TNF superfamily. This study aimed to evaluate the effect of TNFSF14 on the levels of inflammatory cytokines involved in pulpal inflammation using lipoteichoic acid (LTA)-induced human dental pulp stem cells (hDPSCs). hDPSCs were cultured and induced with LTA, followed by treatment with TNFSF14 at 25 and 50 ng/mL. Cellular viability was evaluated using the Alamar Blue assay. The levels of inflammatory cytokines IL-6, IL-8, IL-10, and TNF-α were quantified using reverse transcription–quantitative polymerase chain reaction (RT–qPCR) and enzyme-linked immunosorbent assay (ELISA). TNFSF14 at 25 and 50 ng/mL significantly reduced the mRNA and protein levels of pro-inflammatory cytokines *TNF-α*, *IL-6*, and *IL-8*, and increased the anti-inflammatory cytokine *IL-10*. In addition, TNFSF14-treated groups enhanced cell viability. Adding TNFSF14 to LTA-induced hDPSCs regulated the production of inflammatory cytokines by lowering the levels of IL-6, IL-8, and TNF-α and elevating IL-10 levels.

## 1. Introduction

Dental caries affects about 2 billion individuals around the world, which is equivalent to over one-third of the global population, thereby constituting a major public health concern for both individuals and governments [1,2]. Dental caries is a bacterial infection that, if untreated, will result in dental pulp inflammation and eventual necrosis. The impact of dental caries is mostly related to its effect on dental pulp. where pain is a major manifestation of pulpal disease [3].

The financial burden of this disease is attributed to the direct cost of treatment, as well as lost days of work by affected individuals [4,5]. In addition, dental caries can affect the quality of an individual’s life by interfering with food intake, and causing pain and discomfort, and more recently a link has been suggested between chronic oral disease and systemic diseases, such as diabetes, heart disease, and hypertension [6,7].

Pulp–dentin complex functions as one physiological unit and it is protected by enamel and cementum [3]. Healthy pulp can provide several defense mechanisms against any external noxious stimuli [8]. The initial response to various irritating stimuli is inflammation, followed by tertiary dentine deposition in response to injury. There are two types of tertiary dentin, reactionary and reparative, depending on the extent of the disturbance to the pulp–dentin complex. Reactionary and/or reparative dentin will be formed to protect the pulp and maintain the vitality of the pulpal tissues [9]. In case of mild to moderate insults, tertiary dentine will be secreted and inflammation will be resolved [10]. If the pulp–dentin complex is not able to resolve the insult, such as in the case of prolonged and/or severe irritation, death of odontoblasts will occur and the inflammatory process becomes irreversible and leads, eventually, to pulpal necrosis and periapical lesion formation [3,11].

Inflammation of the pulp–dentin complex results in cellular and molecular events that aim to remove the infection and promote wound healing. Several cell types have the potential to detect any pathogen within the pulp–dentin complex, including odontoblasts, which are the first line of defense and pathogen detection, as well as fibroblasts, immune cells, and human dental pulp stem cells (hDPSCs) [12]. These cells express pattern-recognition receptors (PRRs), particularly Toll-like receptors (TLRs), which can recognize pathogen-associated molecular patterns (PAMPs), such as lipoteichoic acid (LTA), which exists in gram-positive bacteria. TLR activation by LTA will result in the upregulation of cytokines and will bind to Toll-like receptor 2 in case of infected dentin, then the immune response will be initiated, and inflammatory cytokines will be produced [13]. Nuclear factor-kappa B (NF-κB) and p38 mitogen-activated protein (MAP) kinase pathways have been recognized as controls for a wide range of inflammatory mediators. These signaling pathways are activated by the interaction between LTA and TLR2, resulting in the production of inflammatory cytokines. Thus, activation of NF-κB and MAP kinase in these cells is essential for regulating inflammation and tissue repair mechanisms [12,14]. The molecular events, influenced by TLR signaling, NF-κB, and MAP kinase activation, highlight the complicated nature of the pulp’s defense mechanisms against pulpal inflammation.

Cytokines can be divided into pro-inflammatory and anti-inflammatory cytokines. Pro-inflammatory cytokines mediate and enhance inflammation, while anti-inflammatory cytokines generally reduce inflammation. Several inflammatory cytokines are involved in pulpal inflammation, such as tumor necrosis factor-alpha (TNF-α), interleukin (IL) 6, IL-8, and IL10 [15]. Cytokines function by binding to receptors present on multiple cell types which will result in amplification or reduction of the inflammatory response within the tissue [16].

IL-6 is secreted by multiple cell types in reaction to bacterial infections, allowing inflammation and enhancing pro-inflammatory mediators. This enhances the immune response and leads to tissue destruction. Increased IL-6 concentrations in inflamed pulp stimulate matrix metalloproteinases, resulting in the degradation of the extracellular matrix [17,18]. IL-8 plays a significant role in dental pulp inflammation by recruiting neutrophils and enhancing the immune response. In response to infections, IL-8, produced by various cells, results in elevated immune cell infiltration and tissue damage [19]. TNF-α is a critical cytokine involved in dental pulp inflammation, synthesized by multiple cells in response to bacterial infection. It facilitates the recruitment of immune cells and enhances inflammation, leading to tissue damage in inflamed pulp. Increased levels of TNF-α are significantly associated with the degree of pulpal inflammation [20]. Fibroblasts from the dental pulp secrete anti-inflammatory IL-10 to modulate inflammation by suppressing the secretion of pro-inflammatory cytokines, such as IL-6, through the inhibition of the NF-κB pathway, thereby limiting inflammation of the dental pulp [21].

The management of pulpal disease involves the elimination of the causative factor and the application of a material that will facilitate healing of the pulp and the formation of a mineralized dentin bridge. Different materials have been used in the management of pulpal infections to minimize inflammation and facilitate the healing process. Among these materials are calcium hydroxide and calcium silicate-based materials, including Bio-dentine and mineral trioxide aggregate [22].

Although the endodontic profession has made significant progress in terms of materials aiming to reduce inflammation and promote memorized tissue formation, materials currently used in management of the pulpal infection produce early inflammatory responses at different levels in superficial and deep pulpal tissues [23]. In fact, it is common to observe inflammatory cell infiltration, at least in the early stages of placing these materials [24].

Tumor necrosis factor superfamily 14 (TNFSF14), also known as the lymphotoxin-like inducible protein that competes with glycoprotein D for binding to the herpes virus entry mediator on T cells (LIGHT), is an immunomodulatory cytokine member of the TNF superfamily. TNFSF14 is involved in both innate and adaptive immunological responses and promotes the homeostasis of lymphoid organs, bone, and liver [25,26]. The role of TNFSF14 in inflammation is not clearly understood, with pro-inflammatory effects reported under certain conditions and anti-inflammatory effects under others. For example, TNFSF14 has been shown to increase inflammatory responses in adipose tissues in vivo [27]; on the other hand, the presence of TNFSF14 reduces chronic intestinal inflammation in mice [28]. Furthermore, there is evidence to suggest an anti-inflammatory effect of TNFSF14, in which it plays a protective role against certain diseases in vivo [28,29]. When the inflammatory effect of TNFSF14 against interferon-γ stimulated human gingival fibroblasts was investigated, TNFSF14 upregulated proinflammatory chemokines [30]. TNFSF14 regulates essential cytokines involved in inflammation, as demonstrated in studies of various diseases. It stimulates the production of IL-6 and IL-8, which facilitate acute-phase responses and the recruitment of immune cells, while also enhancing IL-10 production, indicating its anti-inflammatory potential in certain situations. Furthermore, TNFSF14 increases TNF-α expression, highlighting its significance in pro-inflammatory signaling. These findings highlight TNFSF14’s significance for regulation of the immune response [31,32].

To date, no published paper has investigated the anti-inflammatory effect of TNFSF14 on hDPSCs; therefore, the aim of this study was to evaluate the effect of TNFSF14 on the viability and the levels of inflammatory cytokines, IL-6, IL-8, IL-10, and TNF-α, using LTA-induced hDPSCs. The null hypothesis that guided this work was that TNFSF14 has no effect on LTA-induced cytokine production by hDPSCs

## 2. Materials and Methods

### 2.1. Overview of the Study Design

The study consisted of four groups: a control group, a group that received only LTA, and two treatment groups that received varying concentrations of TNFSF14. Cellular viability, mRNA levels, and protein concentrations of inflammatory cytokines were evaluated through the Alamar Blue assay, RT–qPCR, and ELISA, respectively. Each testing procedure was conducted blindly by a single trained operator.

### 2.2. Study Groups Allocation

The study involved the following groups: Control Group: hDPSCs cultured in supplemented α-MEM without LTA or TNFSF14; LTA Group: hDPSCs treated with LTA (10 µg/mL) but without TNFSF14; TNFSF14 Treatment Groups: hDPSCs exposed to LTA (10 µg/mL) and treated with TNFSF14 at two concentrations: 25 and 50 ng/mL.

### 2.3. Experimental Setup

#### 2.3.1. Cell Culture

hDPSCs were purchased from Lonza (Basel, Switzerland, catalog number PT-5025) and expanded in alpha-modified minimum essential medium (α-MEM) supplemented with 10% fetal bovine serum (FBS), 1% penicillin–streptomycin, and 1% MEM Non-Essential Amino Acids Solution (NEAA). Cells were cultured in a humidified incubator at 37 °C and 5% CO_2_. For experiments, cells from passages 3–6 were used. Once cells reached 90% confluency, they were transferred to appropriate well plates for experimental procedures.

#### 2.3.2. Reagent and Lipoteichoic Acid Induction of Dental Pulp Stem Cells

Recombinant human TNFSF14 (R&D Systems Minneapolis, MN, USA, catalog number 664-LI-025) was dissolved in 0.1% bovine serum albumin (BSA)-phosphate-buffered saline (PBS) and stored at −20 °C until use according to the manufacturer’s instructions. Purified LTA derived from *Staphylococcus aureus* (InvivoGen, San Diego, CA, USA, CAS 56411-57-5) was applied at 10 µg/mL to induce an inflammatory response in hDPSCs.

### 2.4. Alamar Blue Cell Viability Assay

The effect of TNFSF14 on the viability of LTA-induced hDPCs was assessed at two concentrations, 25 and 50 ng/mL, on days 1, 2, 3, and 7, using the Alamar Blue assay [33]. In brief, cells were seeded with a density of 5 × 10^3^ cells per well in a 96-well plate and kept in an incubator at 37 °C, 5% CO_2_, and 95% humidity. At the end of each time point, the media were removed and replaced with fresh media containing 10% Alamar Blue reagent (Bio-Rad Inc., Hercules, CA, USA). The fluorescence of each plate was measured at an excitation of 530 nm and an emission of 590 nm by a fluorescence reader SpectraMax^®^ M5/M5e Multimode Plate Reader (Molecular Devices, San Jose, CA, USA). Data were acquired using SoftMax^®^ Pro 6 Microplate Data Acquisition and Analysis Software (Molecular Devices). The experiments were conducted in duplicate with nine samples in each experiment (*n* = 18).

### 2.5. Inflammatory Cytokine Levels of LTA-Induced Dental Pulp Stem Cells

#### 2.5.1. Reverse Transcription–Quantitative Polymerase Chain Reaction (RT–qPCR)

hDPSCs were seeded in a 6-well plate in supplemented α-MEM with a density of 5 × 10^5^ cells/well. On the second day, cells were induced with LTA and concurrently exposed to 25 and 50 ng/mL of TNFSF14 for two hours. RNA lysis buffer was added to the wells then RNA was isolated using the RNeasy mini kit (RNeasy; Qiagen, Hilden, Germany), then quantified using a Nanodrop spectrophotometer (Nanodrop 2000, Thermo Fisher Scientific, Agawam, MA, USA). A high-capacity cDNA reverse transcription kit (Thermo Fisher Scientific) was used to reverse transcript the extracted RNA and synthesize cDNA using a multigene cycler (Labnet International, Inc., Edison, NJ, USA). Next, Fast SYBRTM Green PCR Master Mix (Thermo Fisher Scientific) was used to analyze messenger RNA (mRNA) expressions under the following thermal conditions according to the manufacturer: 95 °C for 12 min followed by 40 cycles of 95 °C for 15 s, 65 °C for 30 s, and 72 °C for 30 s. The primer sequences for *IL-6*, *IL-8*, *IL-10*, and *TNF-α* (OligoTM, Seoul, South Korea) are listed in Table 1. After 45 amplification cycles, cycle threshold (Ct) values were obtained and normalized to the endogenous control Glyceraldehyde-3-phosphate dehydrogenase (GAPDH) values. The comparative delta–delta Ct method (2^−ΔΔCT^) was used to normalize the data based on the endogenous reference (*GAPDH*) to be expressed as the relative fold change compared to the values obtained from the LTA only group. Experiments were performed in duplicate [34].

#### 2.5.2. Enzyme-Linked Immunosorbent Assay (ELISA)

hDPSCs were seeded in a 96-well plate in supplemented α-MEM with a density of 1 × 10^4^ cells/well. The following day, hDPSCs were induced with LTA for four hours, and supernatants were collected for the detection of TNF-α, IL-8 IL-6, and IL-10 using Enzyme-linked immunosorbent assay (ELISA) following a standard protocol [35].

Standards were reconstituted according to the manufacturer’s protocol for TNF-α, IL-8 IL-6, and IL-10 assays (Thermo Fisher Scientific, KAC1261; CAS EHIL10; KAC126; BMS223-4). Standards and samples were added to TNF-α, IL-8 IL-6, and IL-10 antibody-coated wells so that antigens bound to their respective adsorbed antibodies. Next, the plates were aspirated and washed. Biotin conjugate antibody was added to the captured antigens by the first immobilized adsorbed antibody. Following aspiration, washing procedures were carried out.

Thereafter, Streptavidin–HRP was added to bind to the biotin conjugate plates and rewashed to remove unbound antibodies, then the chromogenic substrate was added to produce color changes until the solutions turned blue, and then the reaction was stopped by a stop solution.

Absorbance was read by an absorbance reader SpectraMax^®^ M5/M5e Multimode Plate Reader (Molecular Devices) at 450 nm. Data were acquired using SoftMax^®^ Pro 6 Microplate Data Acquisition and Analysis Software (Molecular Devices). The data were normalized to be expressed in fold change compared to the values obtained from the LTA only group. The experiments were conducted in duplicate. Fold change was determined by normalizing the LTA-only group to a value of 1 and calculating the other groups compared to it.

### 2.6. Statistical Analysis

The normality of the distribution was evaluated using the Shapiro–Wilk test. A parametric one-way ANOVA test was performed when the data followed a normal distribution. The Levene test was performed to confirm the equality of variances followed by the Tukey post hoc test. Welch ANOVA with Games–Howell post-hoc tests were applied if the assumption was not observed. A Kruskal–Wallis non-parametric test was conducted on the viability assay values. The results were considered statistically significant if the *p*-value was <0.05. The analysis was conducted using IBM SPSS Statistics software version 29. GraphPad Prism 10 (GraphPad Software, San Diego, CA, USA) was used to design the graphs.

## 3. Results

TNFSF14 at concentrations of 25 and 50 ng/mL promoted cell viability, reduced the mRNA and protein levels of pro-inflammatory cytokines *TNF-α*, *IL-6*, and *IL-8*, and elevated the anti-inflammatory cytokine *IL-10*.

### 3.1. Alamar Blue Cell Viability Assay

**Figure 1:**
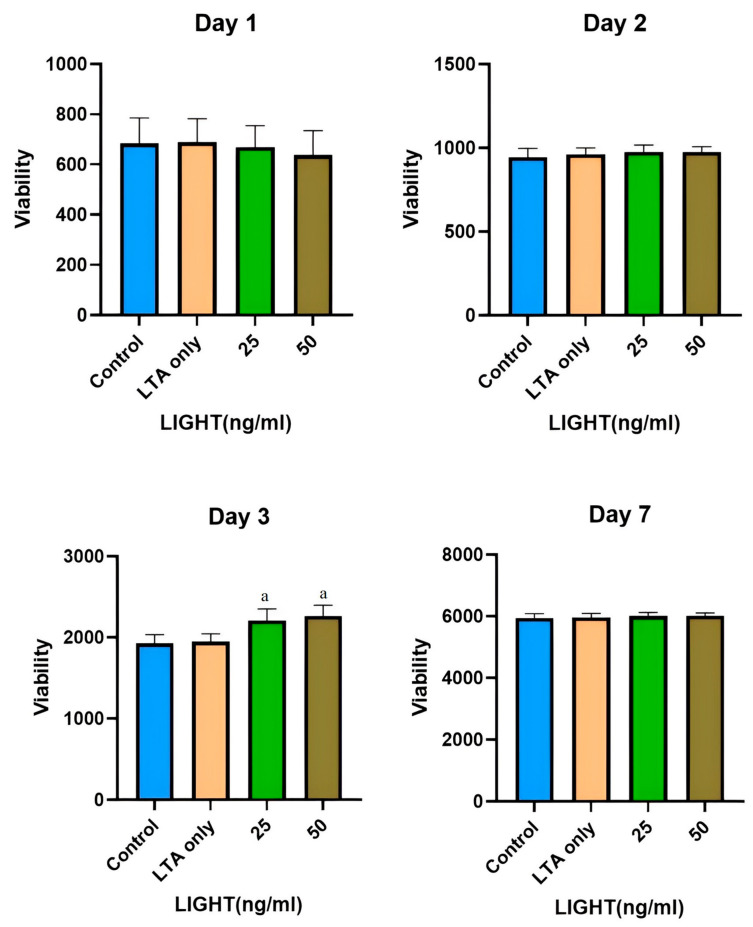
hDPSC viability on Days 1, 2, 3, and 7. Data are shown as mean ± SD. ^a^ Represents a statistically significant difference *p* < 0.001 compared to the control and LTA only groups.

The Kruskal–Wallis test showed no statistically significant differences between groups on days 1 and 2. However, on day 3, the 25 and 50 ng/mL TNFSF14 groups exhibited a statistically significant increase in cell viability compared to other groups (*p* < 0.001). On day 7, the 25 and 50 ng/mL TNFSF14 groups demonstrated greater viability, but this was not statistically significant (Figure 1).

### 3.2. Reverse Transcription–Quantitative Polymerase Chain Reaction (RT–qPCR)

**Figure 2:**
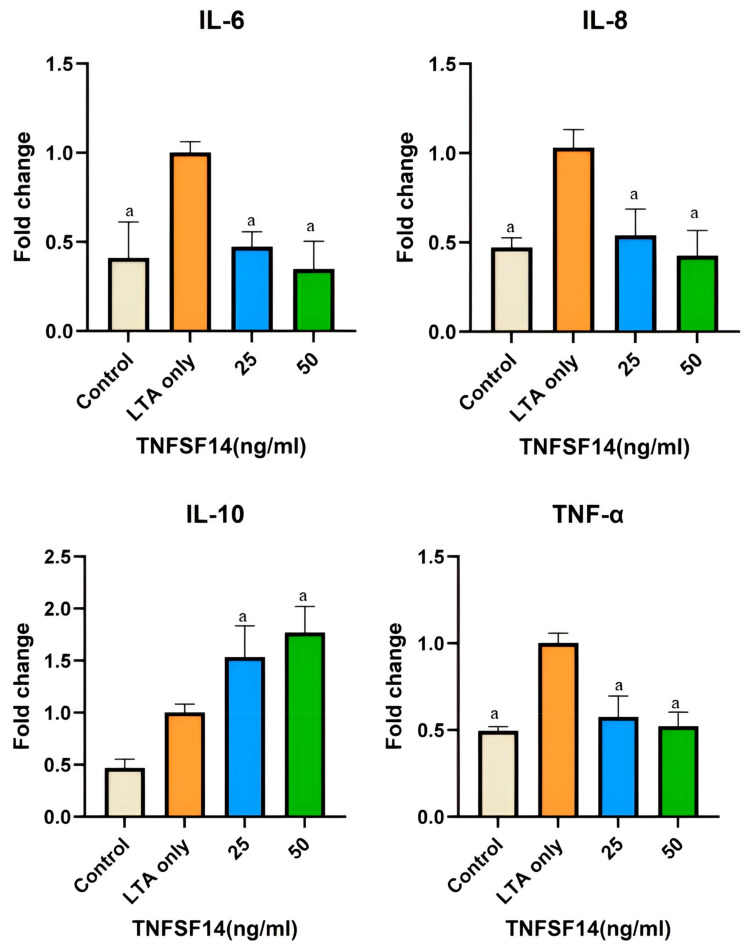
mRNA levels expressed in fold change as measured by RT–qPCR of *IL-6*, *IL-8*, *IL-10*, and *TNF-α*. ^a^ Represents a statistically significant difference *p* < 0.001 compared to the LTA only group.

In LTA-induced hDPSCs, adding 25 and 50 ng/mL TNFSF14 resulted in a significant reduction in the mRNA levels of *TNF-α*, *IL-6*, and *IL-8* (*p* < 0.001). On the other hand, there was a significant increase in *IL-10* mRNA levels in TNFSF14-treated groups compared to the control group and the LTA group. mRNA levels of *IL-10* showed a significant increase in the LTA group compared with the control group (*p* < 0.001) (Figure 2).

### 3.3. Enzyme-Linked Immunosorbent Assay (ELISA)

**Figure 3:**
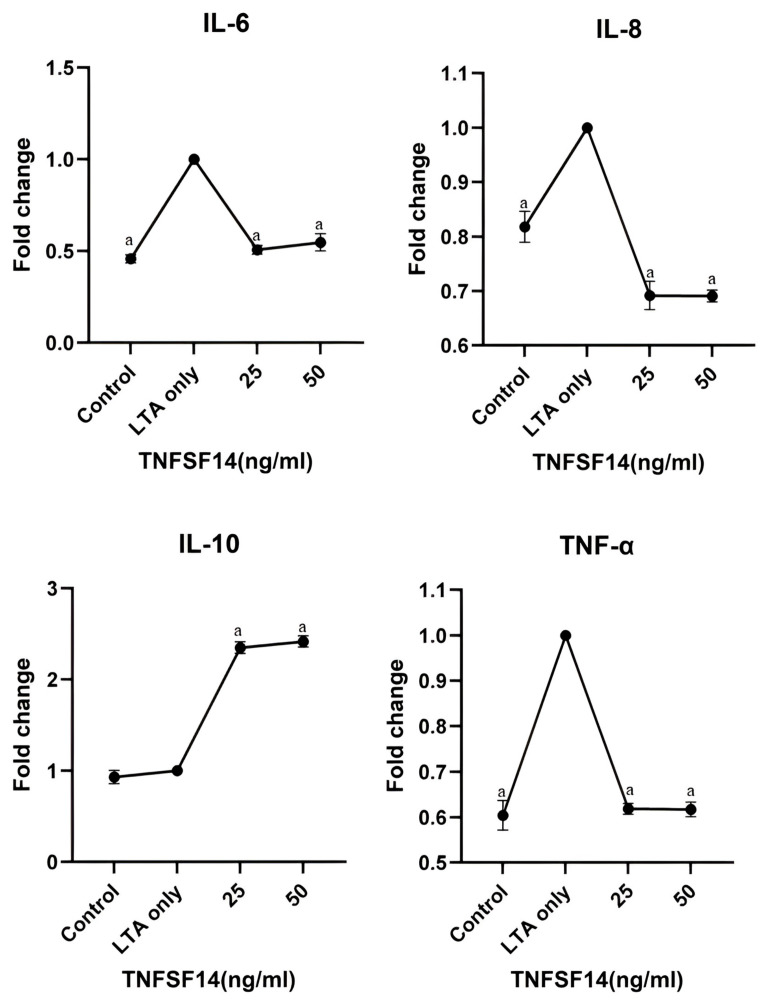
Inflammatory cytokine levels expressed in fold change as measured by ELISA of IL-6, IL-8, IL-10, and TNF-α. ^a^ Represents a statistically significant difference *p* < 0.001 compared to the LTA only group.

Adding 25 and 50 ng/mL of TNFSF14 to LTA-induced hDPSCs resulted in a significant reduction of IL-6, IL-8, and TNF-α levels as measured by ELISA (*p* < 0.001). Conversely, there was a significant increase in IL-10 levels in the LTA-induced hDPSCs treated with 25 and 50 ng/mL of TNFSF14 (*p* < 0.001) (Figure 3).

## 4. Discussion

Gram-positive bacteria are a primary cause of dental caries and pulpal inflammation. Salivary proteins interact with Gram-positive bacteria or, more specifically, its virulence factor LTA, to play a critical role in host immunological responses and bacterial pathogenesis throughout the development and progression of dental caries [36].

This study is the first to evaluate the modulatory effects of TNFSF14 on LTA-induced inflammatory response of hDPSCs. The findings suggest a beneficial anti-inflammatory property of TNFSF14, which has potential in the treatment of inflammatory pulpal diseases.

In particular, the current study revealed a significant reduction in the mRNA levels of pro-inflammatory cytokines *TNF-α*, *IL-6*, and *IL-8* in the groups treated with TNFSF14. Conversely, there was a notable increase in the anti-inflammatory cytokine *IL-10*. These results were supported by the ELISA findings, which showed a similar trend in protein expression levels. Furthermore, cell viability was enhanced in TNFSF14-treated groups. The null hypothesis was rejected because TNFSF14 induced changes in cytokines produced by LTA-induced hDPSCs.

LTA is a particular component of Gram-positive bacteria, acting as an antigen that activates immune cells to initiate immunological reactions. hDPSCs are sensitive to LTA on their cell surface through TLR2. When hDPSCs TLR2 interacts with LTA, it activates TLR2, causing the release of a variety of inflammatory cytokines [13,37]. NF-κB and MAPK pathways are essential for the inflammatory response. It is well established that activating TLR2 by LTA activates the NF-κB and MAPK pathways, producing inflammatory mediators [12].

Cytokines promote communication between odontoblasts and innate immune system cells, such as neutrophils. Both odontoblasts and immune cells produce these proteins in response to bacterial stimuli to attract more immune cells and regulate inflammatory responses [38].

Pro-inflammatory cytokines, such as TNF-α, IL-6, and IL-8, play an important role in the immune response and tissue repair processes of the pulp–dentin complex. Increased levels of these cytokines during pulpal inflammation exacerbate the inflammatory response by attracting immune cells [15,39]. In contrast, anti-inflammatory cytokines, including IL-10, are essential for the resolution of inflammation. IL-10 inhibits the production of pro-inflammatory cytokines. The progression or resolution of pulpal inflammation is determined by the balance between pro-inflammatory and anti-inflammatory cytokines [39,40].

Managing pulpal disease requires eliminating the etiological factors and using materials that promote pulp healing and the formation of mineralized tissue. Various materials have been used to treat pulpal infections, aiming to reduce inflammation and promote the healing process. Notably, calcium silicate-based materials, such as Bio-dentine and mineral trioxide aggregate, are commonly used due to their prominent biocompatibility, bioactivity, and sealing capability [22]. However, materials currently used in management of pulpal infection, such as mineral trioxide aggregate, produce early inflammatory responses at different levels in pulpal tissues [23]. Elevated levels of inflammatory cytokines, like IL-6 and TNF-α, may inhibit the mineralization potential of hDPSCs [41]. Additionally, the upregulation of IL-8 is associated with a decrease in odontogenic markers, such as dentine sialoproteins. Consequently, the entire healing process may be compromised [42].

The reduction in the levels of pro-inflammatory cytokines and the elevation of the anti-inflammatory cytokines produced by TNFSF14 suggest a role of this material in promoting pulp healing following an insult. In fact, inflammation plays a major role in pulpal tissue damage, while its resolution is essential for returning the pulp to the state of normalcy [12]. Since current therapeutic materials used to manage inflamed pulps is associated with inducing inflammation [23,24], TNFSF14 could prove advantageous in the management of pulpal disease, either on its own or as an adjunct to currently used materials.

This study was performed in an in vitro model using a single cell type, which may not completely reproduce the complex physiological and cellular environment of dental pulp. Conducting experiments on other pulpal cell types, as well as using other inducers of inflammation, such as lipopolysaccharides, will add to our understanding of the role of TNFSF14 in dental pulp disease. In addition, testing the material in an in vivo model is essential prior to introducing the material for clinical use.

Dental caries remains a significant worldwide health issue; introduction of TNFSF14 as an alternative therapeutic agent could transform the management of carious lesions associated with pulpal inflammation. TNFSF14 may be involved in developing biomaterials intended to enhance pulpal healing and reduce inflammatory responses, thereby providing an innovative strategy for addressing dental caries and its associated consequences.

## 5. Conclusions

This study offers new insights into the impact of TNFSF14 on LTA-induced hDPSCs, demonstrating TNFSF14’s potential as a modulator of inflammatory responses. The findings of our study demonstrate that TNFSF14 could effectively reduce the inflammatory reaction by decreasing the levels of pro-inflammatory cytokines, including TNF-α, IL-6, and IL-8, while simultaneously promoting the synthesis of the anti-inflammatory cytokine IL-10.

## Figures and Tables

**Table 1:** List of primer sequences for *GAPDH*, *IL-6*, *IL-8*, *IL-10*, and *TNF-α*.

Gene	Sequence (5′-3′)
***GAPDH***	Sense (forward primer) 5′-CTGGTAAAGTGGATATTGTTGCCAT-3′ Antisense (reverse primer) 5′-TGGAATCATATTGGAACATGTAAACC-3′
***IL-6***	Sense (forward primer) 5′-GCCCAGCTATGAACTCCTTCT-3′ Antisense (reverse primer) 5′-GAAGGCAGCAGGCAACAC-3′
***IL-8***	Sense (forward primer) 5′-GGCACAAACTTTCAGAGA CAG-3′ Antisense (reverse primer) 5′-ACACAGAGCTGCAGAAATCAGG-3′
***IL-10***	Sense (forward primer) 5′-TGAGCTTCTCTGTGAACGATTTA-3′ Antisense (reverse primer) 5′-GTCACCCTATGGAAACAGCTTA-3′
***TNF-α***	Sense (forward primer) 5’-GAGGCCAAGCCCTGGTATG-3’ Antisense (reverse primer) 5’-CGGGCCGATTGATCTCAGC-3’

## Data Availability

The original contributions presented in this study are included in the article. Further inquiries can be directed to the corresponding author.
